# Fully synthetic replication of complex real biological cell clusters using a novel cluster-based ‘Rosetta-Routine’ computational modelling process

**DOI:** 10.1371/journal.pcbi.1014280

**Published:** 2026-05-29

**Authors:** Bradley Mason, Laura Justham, Liam Whitby, Alison Whitby, Stuart Scott, Samuel Nti, Jon Petzing

**Affiliations:** 1 Wolfson School of Mechanical, Electrical and Manufacturing Engineering, Loughborough University, Loughborough, United Kingdom; 2 UK NEQAS for Leucocyte Immunophenotyping, Sheffield Teaching Hospital NHS Foundation Trust, Sheffield, United Kingdom; The Chinese University of Hong Kong Faculty of Science, HONG KONG

## Abstract

Flow cytometry (FC) is essential for the precise quantification and characterisation of individual cell populations in a larger heterogenous cell suspension. FC analysis provides a foundation for advanced clinical diagnostics and is a key component in many life-saving therapeutic strategies across a broad range of medical conditions. However, clinical, industrial and research laboratories alike face significant challenges in validating the metrological and biological accuracy of FC data analysis. Due to the inherent relative nature of FC data and the lack of definitive ‘ground truth’ associated with processed biological samples. This study specifically focuses on generating realistic fully synthetic flow cytometry cell clusters and demonstrating their suitability as substitutes for traditional FC data. The inherent model-based heritage of synthetic data enables the robust ability to generate distributionally-equivalent replicate datasets with explicit knowledge of cluster membership for each individual datapoint. Thereby, reducing the uncertainty issues associated with real cluster data and its analysis. This research uses meticulously optimised synthetic cluster-generating benchmarking software to simulate real monocyte clusters. A central component of the protocol is the ‘*Rosetta-Routine*’, a novel codebase which deciphers the statistical properties of real data and translates them into the computational coefficients required to generate accurate cluster-based synthetic replicates. This innovative approach ensures that the synthetic datasets faithfully represent the statistical characteristics of real-world data while retaining the benefits of computational traceability. This approach addresses a critical gap in current practices by enabling the ability to provide a controlled and reproducible validation framework for assessing clustering methods applied to analyse FC data. These features allow the ability to score and subsequently enhance the analysis confidence in many FC applications such as in diagnostics or in ‘mock-up’ training scenarios. Future synthetic-data-driven enhancements in FC analysis confidence will translate into more accurate clinical decision-making and subsequent overall improvements in patient care.

## Author summary

In this study, we introduce a new method for generating realistic synthetic flow cytometry cell clusters. We utilise a series of robust algorithms and modelling functions to accurately translate data properties from real sample cell clusters into a cluster generator to computationally generate a synthetic replication of the real cluster. The approach demonstrates statistical and visual similarities between the synthetic and original real complex biological clusters, with close alignment in the forward and side scatter graph axes often crucial for initial cell characterisation in flow cytometry. These results display promising future applications in flow cytometry analysis with an end goal of helping to facilitate more consistent diagnostics in clinical and industrial settings. Our paper contributes to the field by offering a unique method for generating synthetic data which statistically mirrors real-world measurements. Thereby providing novel opportunities to evaluate manual and automated cluster analysis methodologies.

## Introduction

Modern flow cytometry is considered the current state of the art ‘gold standard’ for high-throughput single-cell analysis of cellular suspensions in clinical and industrial environments [1,2]. When examining data, it is beneficial to identify the individual subset populations and patterns (clusters) within the data to gain vital diagnostic insights.

The cluster analysis (clustering) of flow cytometry data is frequently carried out manually via operator observational ‘gating’ of two-dimensional pair-wise graphs in specialised analysis software. This analysis is often applied methodically throughout multiple dimensions concurrently to refine a target cell population of interest [3,4]. Yet, these manual gating strategies rely upon human operator judgement, and as a result are prone to the common pitfalls associated with human-based analysis [5,6]. Results naturally differ between individual operators, whether this be due to unavoidable human input uncertainty, unintentional bias, methodology or training differences [7,8].

Automated clustering algorithms were designed to aid and, in some cases, replace manual clustering techniques [9]. Clustering algorithms are tools that extract a hierarchy from low or high-dimensional flow cytometry data; often in an unsupervised manner and allows users to visualise, isolate and quantify multiple cell types without requiring the user to define a known cellular ordering [10].

There is a large abundance of unsupervised and supervised clustering algorithms available. Previous reviews have identified over 50^+^ unique algorithms produced between 2008 and 2019 alone, with an increasing rate in the number of published papers since [11]. Each individual clustering algorithm is consistent in how it analyses data from different datasets but comparing results between different clustering algorithms often demonstrate disagreements in the results between them [12].

Each clustering algorithm employs its own distinct mathematical methods and techniques, which can be either similar or markedly different from one another. These algorithms are introduced in the literature with varying levels of specialisation but are often pitched to fit a wide range of clinical applications simultaneously [13]. However, the general applicability of these algorithms and accuracy of clustering results for an everyday user’s clinical data remains uncertain, especially for specific scenarios not covered by an algorithm’s source material. This underrepresentation in clinical scenario diversity has driven the publication of numerous flow cytometry cluster evaluation studies, offering guidance to operators to better select the most effective clustering algorithms for a specific clinical application [14–16]. In spite of this, the assessment of these clustering algorithms is often achieved by the application of differing metrics to report the performance of clustering data [17–19]. Increasing the analytical convolution and subsequent difficulties in drawing meaningful and user-relevant comparisons between studies.

This persistent influx of clustering algorithms with varying results and assessment metrics has led to questions of how to reliably validate and compare the clustering analysis output for each in individual user-relevant scenarios. The routine sharing of raw data to facilitate such assessments as a solution is highly impractical due to confidentiality concerns and inconsistencies in the data quality. Consequently, there is a growing need for a controlled, ethically robust method to validate data analysis output across both manual and automated clustering solutions in a wide range of clinical applications, enabling standardised and comparable evaluations to be drawn irrespective of the gating or subsequent assessment methods applied.

In flow cytometry, the accuracy of a clustering algorithms’ performance in real data sets is often determined through the comparison of the automated clustering results compared to the ‘true’ values. When necessary, and in the absence of a certified standard, these ‘true’ values can be derived through various methods, such as statistical formulations, scarce cellular certified reference materials, manually gated analysis results from expert laboratories, or consensus manually gated values from both expert and non-expert participants, typically in the form of an accredited external quality assessment scheme [20–23]. However, the inherent variability among biological lab processes and operators ubiquitously present in these approaches also invite sub-optimal uncertainty-induced clustering outcomes and potential biases [24].

The rapid influx of multiple clustering algorithms with novel approaches inevitably led to the creation of further computational processes that could be used to benchmark and assess the capabilities and differences between these clustering algorithms. With these benchmarking processes occurring under a completely user-controlled synthetic environment, thereby removing the cluster population uncertainties present in the real data. Synthetic cluster generators were initially conceived and designed as usage-agnostic ‘sandbox’ implementations to create user-designed cluster data. However, these synthetic cluster generators are being increasingly utilised in clustering algorithm benchmarking purposes; as an alternative approach to directly using real data to quantify and test the effectiveness of clustering algorithms compared to one another [25,26].

These synthetic cluster generators are a lot less common than the clustering algorithms themselves, with approximately 15^+^ primary iterations openly published and available for use since 1985 [25–39]. Cluster datasets generated using a cluster generator exhibit a superior analytical model free from the random biological and operator uncertainty associated with real data. However, while extremely powerful and useful, many of these cluster generators fall short in one or more aspects required to accurately replicate real flow cytometry cluster data such as higher dimensionality requirements, variance complexity, density control, overlap control and shape control. Furthermore, current cluster data created utilising conventional generators typically assume well-separated, simple Gaussian clusters with limited skew and minimal tail mass; by contrast, flow-cytometry data commonly display asymmetric distributions, overlapping populations and biologically meaningful but sparsely populated subclusters. As a result, data currently generated synthetically via a cluster generator often falls short of the complexity and independent feature controls required to act as a suitable surrogate for complex high-dimensional real flow cytometry cell data.

In summary, real cell data is inherently subjected to uncontrollable factors and as a result pose challenges for accurate estimation. Synthetic datasets provide a controlled and adjustable alternative that could ‘step in’ in conjunction with standardised materials to provide a controlled reference framework for assessing clustering accuracy in the current web of uncertainty surrounding flow cytometry data analysis. Providing a methodology to generate synthetic cluster data of the desired high-complexity akin to real data is established.

User-controlled generation of synthetic flow cytometry datasets offers a powerful route to reduce the cluster population ambiguity prevalent in real traditional datasets, while providing reproducible, precisely parameterised cluster data. Such synthetic flow cytometry data would serve a dual purpose in the flow cytometry community. Firstly, as an unbiased analytically superior performance evaluation method between clustering and gating algorithms, but more importantly acting as a programmable tool to ‘poke’ and expose common failure modes in both automated and manual clustering analysis.

Carefully designed synthetic datasets could emulate challenging or rare scenarios (for example, paediatric phenotypes, instrument faults such as sudden laser drop or Photomultiplier drift, or low-frequency cell populations), and permit scenario-based stress-testing of analysis methods, operator training and quality assurance procedures. The resultant quantitative feedback would help enhance overall analytical consistency and ultimately bolster confidence in flow cytometry analysis. Particularly given that real samples to achieve this may not always be available or accessible in every setting due to scarcity of samples or ethical reasons. Because the synthetic samples are labelled and parameterised, they also enable quantifiable assessment of operator variability, objective measures of gating uncertainty and systematic mapping of algorithmic failure modes (split/merge errors, sensitivity to overlap, etc.). Such user-controlled synthetic data could be seamlessly integrated alongside wet samples into external quality assessment schemes, further advancing consistency in analyses across laboratories.

Central to the utility of such synthetic data is the faithful preservation of the original cluster geometry: relative positions, density gradients and boundary overlap encode biologically meaningful signals (heterogeneity, transitional states, and gating thresholds) that analytic and clinical workflows rely upon. Synthetic clusters that do not retain these relationships will fail to reproduce the analytical challenges of real data (for example, overlapping boundaries that cause gating ambiguity or asymmetric spreads that mask rare events) and thus will provide misleading or incomplete validation. To fulfil these complex criteria, our ensuing methodology synthetically generates data at the individual cluster-by-cluster level to enable a much sought after heightened resolution of controllability and intricacy when creating whole datasets utilising multiple synthetic clusters. The end goal of this bespoke end-to-end, cluster-by-cluster generation strategy is to deliver a high resolution of controllability and realism, enabling labelled, parameter-defined datasets for algorithm benchmarking, regulatory ‘digital reference’ samples, reproducible training sets, and machine-learning augmentation with exact *in-silico* cross-validation.

It is important to note that the present work focuses on the high-fidelity reconstruction of individual clusters as a foundational step. While this enables detailed evaluation of distributional and intra-cluster properties, it does not directly assess inter-cluster discrimination performance (e.g., classification accuracy or Type I/II errors). Extension of this framework to multi-cluster datasets is an important area of ongoing work.

This paper provides the initial framework to synthetically generate clusters quasi-identical to real complex biological flow cytometry cell clusters, with the individual cluster populations defined with explicit and controlled statistical parameters. The fully synthetic data generation reported here is achieved through meticulously employed quantitative methodologies. These combined methodologies analyse the appropriate variables of the original data to extrapolate the statistical parameters required to generate a fully synthetic replicate of this real data with similar statistical, structural and distributional properties, but with data values and visuals dissimilar to the original real data.

## Results and discussion

CluGen is a code program which uses a meta-algorithmic structure, employing support lines (similar to its generateData predecessor [37]) to generate synthetic clusters [39]. The built-in ease of customisation functionalities of CluGen enable the use of bespoke distribution functions and processes. Adapting and expanding the base program’s functional ability to accurately generate realistic flow cytometry-based ‘cell-like’ clusters.

**Fig 1:**
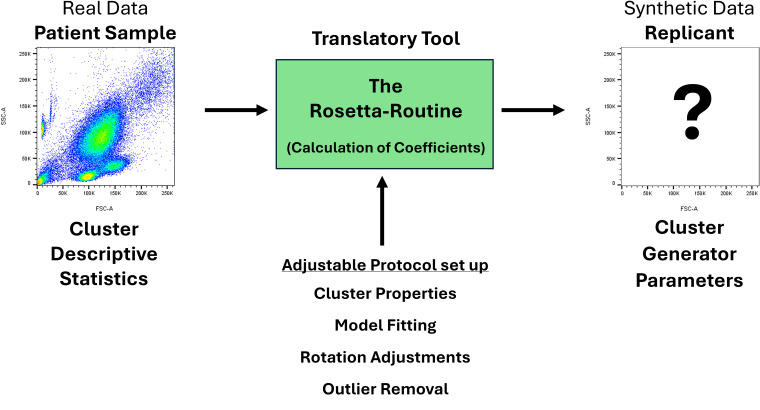
The Rosetta-Routine is the process responsible for converting traditional descriptive statistics into input variables for the cluster generator parameters. Several aspects of the process such as cluster properties, modelling and processing are customisable to provide a greater flexibility in analytical approaches.

The Rosetta-Routine is a vital and novel coded process within the cluster replication methodology which enables high-fidelity computational representations of real individual patient-derived CD34^+^ haematopoietic stem cell enumeration flow cytometry clusters. The Rosetta-Routine, as its namesake suggests, is the translatory tool tasked with deciphering the statistical and distributional properties of real data. Converting these properties into the usable computational coefficients required to replicate the real data synthetically in the cluster generation software, Fig 1. The Rosetta-Routine achieves this by integrating established statistical techniques into a purpose-built pipeline for flow cytometry, with its novelty residing in an end-to-end process that generates synthetic clusters while faithfully preserving the higher-order biological geometries of the original cluster data.

The calculated coefficients for the optimally fitted model required to replicate the original cluster data in the synthetic cluster generator were converted into argument variables for the relevant function measures in the CluGen software, Table 1.

**Table 1:** Mapping of traditional descriptive statistical measures to the conversion methods utilised within the Rosetta-Routine modelling algorithm to acquire the required information from unknown data and the subsequent corresponding cluster generator argument variables.

Descriptive statistics	Rosetta-Routine	Cluster generator
Total number of datapoints	Number of events	*num_points*
Number of dimensions	Number of dimensions	*num_dims*
Number of clusters	Number of clusters and/or components	*num_clusters*
Principal axis orientation	Rotation (°) of maximum variance clockwise from the Vertical	Direction
Range	Modulated by the other variables	Length
Skewness	Skewness (Modified Skewed Gaussian distribution)	*Proj_dist_fn*
Standard Deviation	Standard Deviation of cluster and/or components	*llateral_disp*
Kurtosis	Kurtosis (Modified Kurtosis Gaussian distribution)	*Point_dist_fn* and *llateral_disp* / length ratio
Centroid (Geometric mean)	Dimension-Specific Mean	*Clucenters_fn*
Cluster separation and Overlap	Modulated by the other variables (Verified with Silhouette Score)	Absolute cluster positions (*Clucenters_fn*) and the standard deviation (*llateral_disp*).

### Replicating synthetic clusters

**Fig 2:**
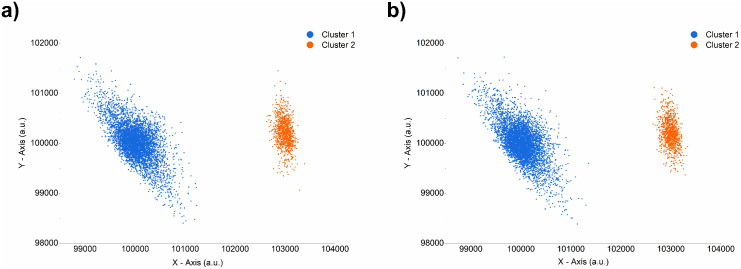
Accuracy testing of the cluster replication process. The original synthetically generated test clusters with known parameters **(a)** and the replicant synthetic clusters generated from the modelling analysis derived parameters **(b)**. Axes represent arbitrary units (a.u.).

To test the initial accuracy of the live cell cluster replication process, controlled synthetic clusters with known properties were manually generated and subsequently analysed with the Rosetta-Routine. The modelling output parameters derived were then used to recreate the original synthetic cluster within CluGen. This synthetic cluster replicant was subsequently assessed to determine the accuracy of the recreation. Success in this initial investigation with ‘cell like’ clusters of a similar total number of datapoints (events) to our smaller real latter cluster samples, containing between 0 – 100,000 events, demonstrates a sound method which can be applied to the real data containing unknown variables. Fig 2, shows the initial manually defined test synthetic clusters(Fig 2a),and the replicant synthetic clusters recreated from the parameters derived from the Rosetta-Routine modelling process (Fig 2b), but generated with a different numerical randomisation seed value. Table 2 depicts the manually defined parameters utilised to generate the original synthetic cluster dataset (Fig 2a), While Table 3 depicts the Rosetta-Routine-derived parameters used to replicate the original synthetic data (Fig 2b).

**Table 2:** Manually defined parameters used to generate the original synthetic cell-like clusters. Parameters were defined a priori to control cluster shape, spread, orientation, and event number, producing synthetic clusters with known properties. These values serve as the ground-truth reference for subsequent replication and accuracy assessment using the Rosetta-Routine modelling pipeline.

Cluster:	Cluster 1 (Blue)		Cluster 2 (Orange)	
**Events:**	5000		1000	
**Rotation (degrees):**	325.00		350.00	
**Dimension:**	**X - Axis**	**Y - Axis**	**X - Axis**	**Y - Axis**
**Mean:**	100,000	100,000	103,000	100,200
**Standard Deviation:**	200	400	100	250
**Skew:**	0.100	N/A	-0.050	N/A
**Kurtosis:**	N/A	0.400	N/A	-0.300
**Silhouette Score:**	0.824			

**Table 3:** Rosetta-Routine-derived parameters used to replicate the synthetic cluster dataset. These derived parameter values were used as inputs for the cluster generation software to create the synthetic cluster replicant dataset. Comparison of these parameters with those in Table 2 enables initial quantitative assessment of the accuracy and fidelity of the cluster replication process.

Cluster:	Cluster 1 (Blue)		Cluster 2 (Orange)	
**Events:**	5000		1000	
**Rotation (degrees):**	325.61		352.08	
**Dimension:**	**X - Axis**	**Y - Axis**	**X - Axis**	**Y - Axis**
**Mean:**	100,007	100,009	102,996	100,204
**Standard Deviation:**	196	398	100	260
**Skew:**	0.096	N/A	-0.049	N/A
**Kurtosis:**	N/A	0.449	N/A	-0.286
**Silhouette Score:**	0.823			

As demonstrated previously, the replication methodology is successful in extracting and defining the parameters needed to create CluGen generated replicates of unlabelled synthetic symmetrical ‘cell-like’ clusters. With the original synthetic cluster generated exhibiting user-specified parameters, while the replicate synthetic clusters were generated with Rosetta-Routine-determined parameters derived from analysing the original unlabelled synthetic test data.

Small identically generated synthetic clusters (<1,000,000 events),with differing Random Number Generation (RNG) seed values, will likely observe differences in the Mean, Standard deviation and higher moments such as Skewness and Kurtosis. This is often due to the statistical moments not reaching adequate stability and achieving statistical convergence with the targeted values during cluster generation. This generational uncertainty was investigated to ensure multiple replicants of the same cluster could be accurately replicated multiple times. These replicates would all display a statistically similar distribution but with differing numerical event points in each, leading to different visual ‘looks’. Multiple replicates of the previous replicant cluster above were generated with differing RNG seeds and analysed, with Table 4. Below displaying the overall mean and uncertainty from the target values calculated for each cluster parameter. The differences observed are insignificant when considering the magnitude of natural variation and spread within identical cell clusters populations between live cell samples in flow cytometry.

**Table 4:** Determining the generational variance between multiple identically generated clusters with differing random number generation seed values. Resultant clusters displayed minor variation between seed values when analysed with the Rosetta-Routine model fitting algorithm. The table displays the Overall Mean results calculated for each parameter along with an error of ± 1 Standard Deviation, (n = 13).

Cluster:	Cluster 1 Replicates		Cluster 2 Replicates	
**Events:**	5000		1000	
**Rotation (degrees):**	324.37 (± 0.72)		351.50 (± 0.61)	
**Dimension:**	**X - Axis**	**Y - Axis**	**X - Axis**	**Y - Axis**
**Mean:**	100,022 (± 4)	100,019 (± 5)	102,990 (± 4)	100,203 (± 7)
**Standard Deviation:**	191 (± 2)	407 (± 4)	99 (± 2)	261 (± 10)
**Skew:**	0.088 (± 0.007)	N/A	-0.046 (± 0.011)	N/A
**Kurtosis:**	N/A	0.404 (± 0.030)	N/A	-0.214 (± 0.065)
**Silhouette Score:**	0.823 (±0.002)			

Dual-pass perpendicular analysis using the Rosetta-Routine can successfully replicate previously generated CluGen clusters with a high accuracy and consistent reproducibility. However, generating accurate replicates of real complex flow cytometry data is more challenging with multiple clusters to consider. Requiring further innovative and novel solutions. This additional complexity in modelling real data compared to the previous synthetic data is due to aspects such as non-symmetricality, superimposition, complex geometric morphology and inter-dimensional correlations.

### Replicating real complex flow cytometry cell clusters

The following refinement protocols along with the application of dual-analysis methodologies, using the Rosetta-Routine, and subsequent generational clustering techniques have been implemented to bridge the gap between the differing requirements and features of modelling synthetic data versus real flow cytometry cluster data.

#### Replicating clusters from datasets containing high levels of superimposition.

**Fig 3:**
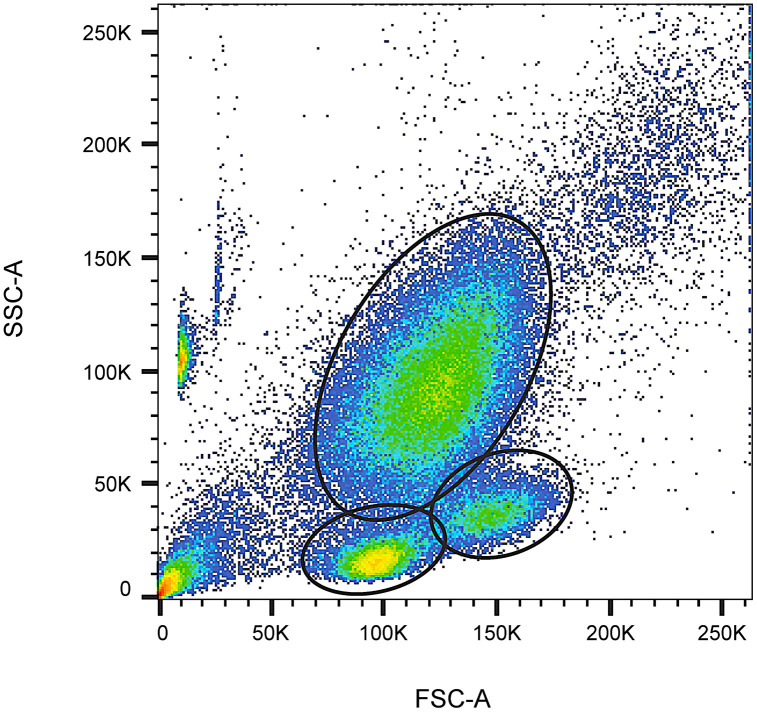
Pseudocolour dot plot of a complete Forward Scatter dimension (FSC-A) and Side Scatter dimension (SSC-A) in CD34^+^ Haematopoietic enumeration analysis demonstrates cluster overlap and associated uncertainties.

Synthetic clusters are often generated at a user’s discretion to contain a large geometric separation or are generated individually so every point, even in overlapping clusters is known. Real flow cytometry clusters on the other hand are all created from biological protocols simultaneously and will usually be in very close proximity to each other or contain a degree of unknown and uncontrollable cluster overlap, Fig 3. Datasets with high levels of superimposition (data points from multiple clusters overlapping significantly) pose unique challenges for accurate cluster modelling. Replicating clusters from such datasets require refinement methodologies to confidently disentangle overlapping distributions and preserve meaningful valid clusters.

The Replication methodology is designed so that individual clusters within a whole dataset can be highly and uniquely customised during cluster generation. This resolution of flexibility requires each cluster to be its own individual model, even in cases of superimposition. To achieve this, the methodology employs a manual core-clustering approach.

Manual ‘gating’ is used as an expert-informed strategy to isolate biologically meaningful core regions of clusters in the joint forward scatter and side scatter space, where boundaries are inherently ambiguous due to overlap and continuous population transitions. In this context, ‘gated’ populations are not treated as absolute sample ground truth, but rather as a consistent reference for statistical modelling. The objective is to reconstruct the underlying distributional structure of the observed data under controlled conditions. In this sense, the current model parameter-driven approach, rather than traditional flow cytometry absolute boundary-driven approaches, aims to leverage and balance the strengths of expert-guided data selection and reduce the impact of operator bias through robust parameter-based reconstruction. Resulting in the establishment of a strong cluster-based foundation for subsequent modelling utilising the Rosetta-Routine.

Manual core-clustering focuses the analysis on the central region of a cluster, excluding the outer overlapping sections of the cluster to eliminate the associated uncertainties. This simplification in data selection enables accurate Rosetta-Routine processing to occur, but subsequent synthetically generated models are truncated with a lower overall density and possible Skew or Kurtosis discrepancies. These inaccuracies due to unavoidable core-clustering of the real cluster data must be reversed to accurately reconstruct and generate synthetic clusters that preserve the full characteristics of the real overlapping original data.

Standard deviation scaling increases the truncated synthetic cluster to the correct size using a scale factor calculated by determining the difference between the standard deviation of the gated real cluster and an identically gated synthetic replicant of the real cluster analysed using the Rosetta-Routine.

The percentage difference between the standard deviations of the real gated and synthetic gated cluster was then doubled (to account for the inherent bimodal modelling of CluGen) and applied to the original x and y standard deviations calculated to generate the original synthetic model. This resulted in a standard deviation adjusted synthetic cluster model comparable to the original cell cluster, Table 5.

**Table 5:** Model output results before and after applying standard deviation scaling. Dimensional-independent standard deviation (SD) scaling between the real and synthetic gated clusters results in a synthetic monocyte cluster gated model with a standard deviation directly comparable to the original real monocyte gated cluster.

Rosetta-Routine output result	Real monocyte cluster gated	Synthetic monocyte cluster ungated	Synthetic monocyte cluster gated	Synthetic monocyte cluster gated (SD adjusted)
**X-Axis Mean:**	146,770	146,875	147,224	147,627
**X-Axis Standard Deviation:**	11,952	12,047	10,806	11,965
**X-Axis Skew:**	-0.025	-0.018	-0.028	-0.027
**X-Axis BIC:**	117,725	117,941	109,500	103,144
**Y-Axis Mean:**	37,142	37,377	37,310	37,388
**Y-Axis Standard Deviation:**	6062	6197	5609	6121
**Y-Axis kurtosis:**	-0.123	-0.125	-0.055	-0.038
**Y-Axis BIC:**	241,014	240,319	225,247	212,593
**X and Y Axis Full Model BIC:**	123,539	123,564	115,896	109,504

**Fig 4:**
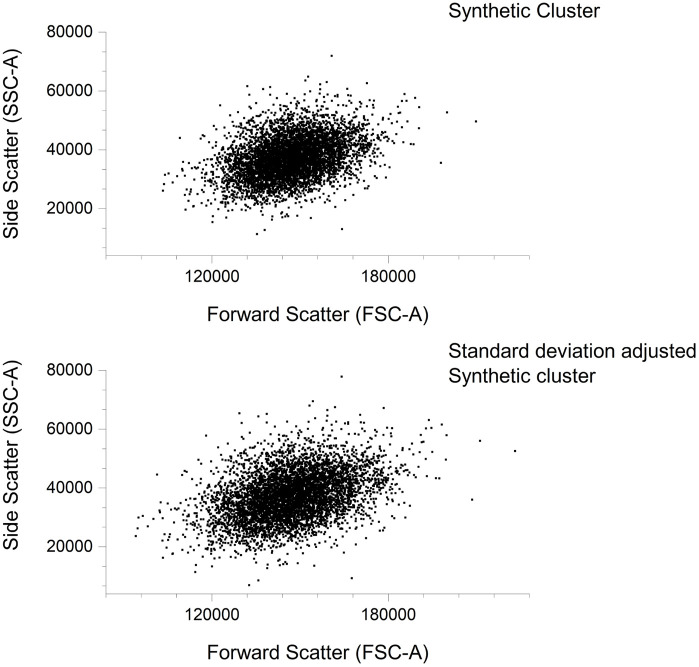
Standard deviation adjustment of the initial synthetic monocyte cluster. The size of initial synthetic cluster was increased by 21.20% in the x dimension and 16.02% in the y dimension. This increase ensured gated standard deviations of the synthetic cluster model were similar to the real gated monocyte cluster.

Fig 4 shows the truncated cluster before and after standard deviation scaling adjustments. With successful resizing of the cluster to match the real cluster, while eliminating the uncertainty caused by the superimposition of the real clusters.

Further modifications were necessary to counteract the distortion in Skewness and Kurtosis induced by the determined and applied increase in standard deviation within the cluster. To address this, scaling factors for each dimension were computed based on the ratio of the updated standard deviation to the original value. Recognising that Skewness is inversely proportional to the cube of the standard deviation, the corresponding Skewness scaling factor was obtained by raising the Standard Deviation scaling factor to the third power. In a similar manner, because Kurtosis is inversely proportional to the fourth power of the standard deviation, the Kurtosis scaling factor was derived by raising the Standard Deviation scaling factor to the fourth power. Consequently, the original Skewness and Kurtosis values were adjusted preserving the true measures of Skewness and Kurtosis despite the changes in Standard Deviation.

The core-clustering of the real data cluster required in datasets with high levels of super imposition results in the total number of data points attributed to the synthetic cluster to be smaller than required. This is due to the truncation of the real cluster’s distribution ‘tails’ and the points within them not being included in the synthetic clusters model.

Density adjustments made via corrections to the number of points in a synthetically generated cluster due to unavoidable core-clustering can be made by comparing the total number of points within the identical gates of the standard deviation adjusted synthetic model and the original real cluster model and increasing the datapoint population to match.

**Fig 5:**
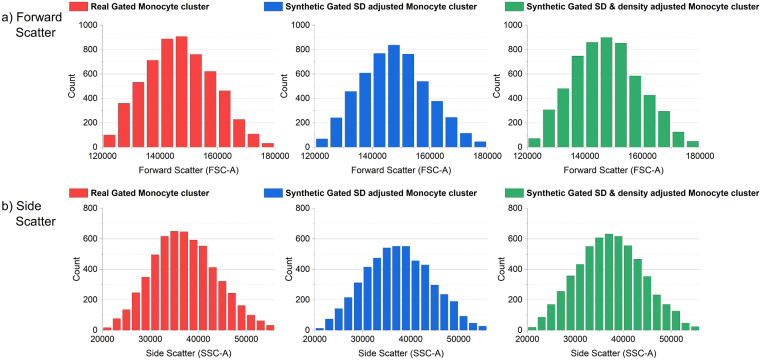
Comparisons between the gated cluster populations in the Forward Scatter and Side Scatter dimensions of real and synthetic monocyte clusters. Comparisons between the total gated datapoint populations in the Forward Scatter (a) and Side Scatter (b) dimensions of real and synthetic monocyte clusters. Both standard deviation and density adjustments together reverse the core-clustering-induced total cluster data point abridgment between the real and synthetic replicate cluster.

In the example, Fig 5 demonstrates the approximate matching of the number of points within the gated real monocyte cluster to the gated synthetic cluster. An increase of 12.8% in the total number of datapoint events in the synthetic cluster was determined by comparing real data with the CluGen generated synthetic adjusted data.

Synthetic data in CluGen is generated using two clear and distinct mirroring unimodal distributions located above and below the cluster support-line (Symmetrical centre line). However real data is deemed multimodal, often with noise or complexity that does not adhere to viable central line splitting with a near-perfect mirroring of distributions.

The bi-modal mirroring nature of synthetically generated CluGen data, offers simplicity, but our current implementations limit the cluster to only a single direction of Skew and/or Kurtosis in one of the axes, which is currently achievable with custom-designed Skew and Kurtosis encompassing distribution functions. On the other hand, real cytometry cluster data is often non-symmetrical in nature due to biological or experimental variability between measurements and will exhibit a different overall direction and/or magnitude of Skew and Kurtosis in every dimension.

However, CluGen can be optimised and its inherent modelling limitations overcome to enable the generation of ‘realistic’ cell clusters. This can be achieved by the novel stacking of two individual CluGen-generated clusters with separate varying perpendicular Skewness and Kurtosis present in the X-axis (“X “cluster) and Y-axis (“Y” cluster).

**Fig 6:**
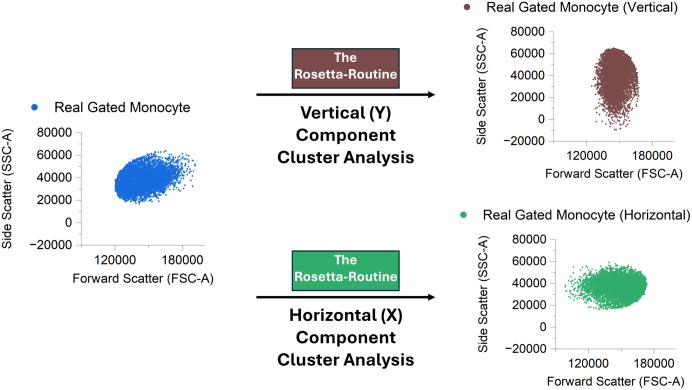
Vertical orientation of the original manually gated Monocyte cluster’s major and minor principal axes. The original manually gated Monocyte cluster is vertically orientated in-line with its respective Minor (X) principal axis (green) and its major (Y) principal axis (Brown). These differing orientations are individually analysed with the Rosetta-Routine to calculate the replication parameters for the individual X-axis and Y-axis components of the original Monocyte cluster.

These processes can be incorporated into the Rosetta-Routine analysis workflow by analysing the original real cell clusters using a dual-analysis approach. The original cluster is orientated to the vertical and horizontal positions using the angle_method (:Vertical and:Horizontal) options. Each orientation is then analysed with the Rosetta-Routine separately (Fig 6) to obtain two separate sets of cluster generator input variables.

This individual dimensional-based component analysis enables the generation of two different interpretations of the original cluster, with one based on the X-axis distribution (X-cluster) and one based on the Y-axis distribution (Y-cluster).

The Rosetta-Routine rotating the original cluster so that its major axis aligns with the horizontal and vertical directions is similar to the initial stages of Principal Component Analysis (PCA). In PCA, the data is rotated such that the directions of maximum variance (the principal components) become the new coordinate axes.

**Fig 7:**
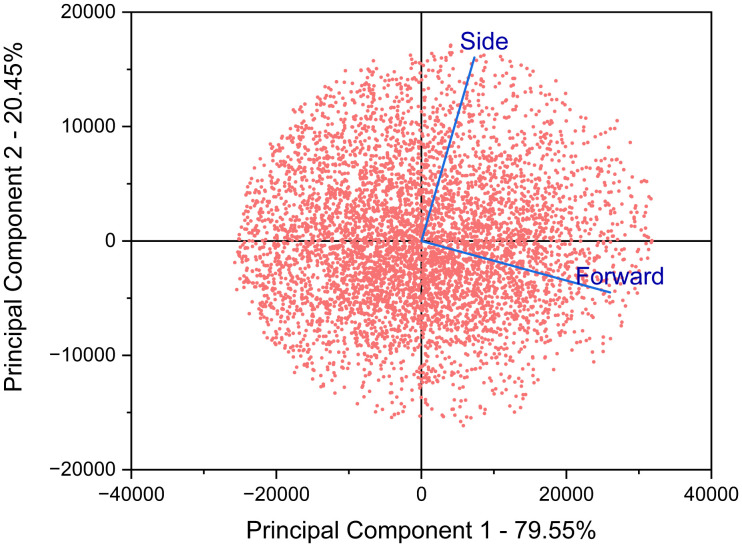
Principal component analysis (PCA) enables variance-based data point allocation between the X and Y component clusters used to generate a synthetic Monocyte cluster. PCA deems Principal component 1 (X-axis) to be responsible for 79.55% of distribution variance in the original cluster, while Principal component 2 has been attributed 20.45% of the total variance.

In PCA, the covariance matrix is decomposed into eigenvalues and eigenvectors. The eigenvalues indicate the amount of variance captured along each principal component, and the eigenvectors define the corresponding directions Fig 7. To generate a synthetic replicate cluster that preserves the variance structure of the original, points are distributed along each principal component in proportion to the variance it explains. Furthermore, the skewness and kurtosis in each dimension are scaled relative to their variance so that the overall shape and higher-order moments of the original cluster are maintained.

**Fig 8:**
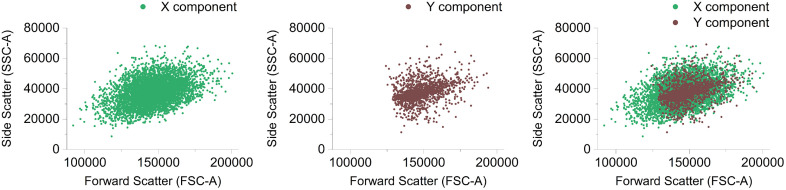
The Dual-analysis Rosetta-Routine approach enables the creation of two separate interpretations of the original Monocyte cluster. Each generated component encapsulates the primary distribution properties of its origin dimension. The dimensional cluster components are rotated in-line with the original clusters angle of rotation and overlayed to create a complete synthetic monocyte cluster with a complex ‘realistic’ distribution.

Each generated component encapsulates the primary distribution properties of its dimension. The dimensional cluster components are rotated in-line with the original clusters angle of rotation and overlayed to create a complete synthetic monocyte cluster with a complex ‘realistic’ distribution, Fig 8.

**Fig 9:**
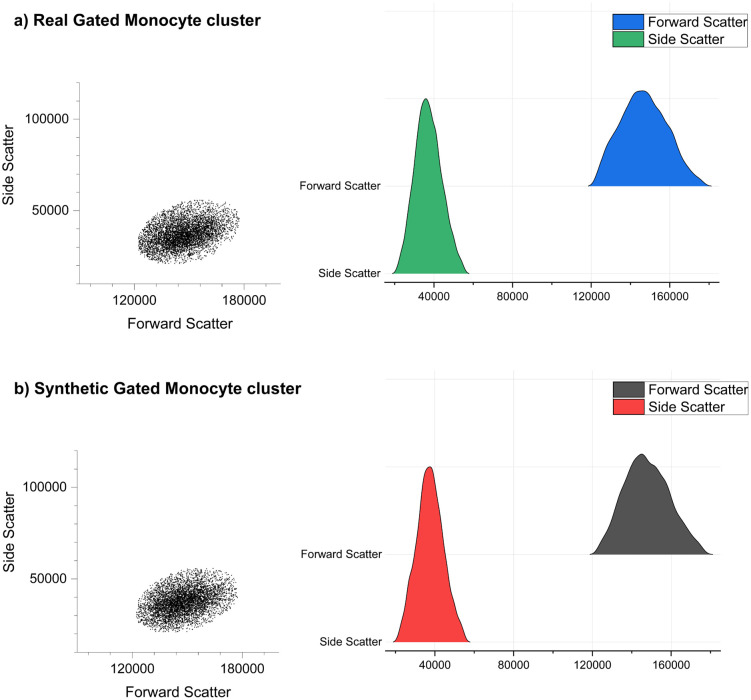
The Dual-analysis approach successfully generates accurate replicates of real Monocyte clusters. The dotplot and ‘kernel smooth’ histogram ridgeline plots for the Real Gated Monocyte Cluster (a) and Synthetic Gated Monocyte cluster (b) enunciates the visual and distributional similarities between each dataset.

Fig 9 Compares the original gated real monocyte cluster and the final synthetic gated monocyte cluster replicant generated after undergoing dual Rosetta-Routine analysis and the subsequent processing steps described above.

**Fig 10:**
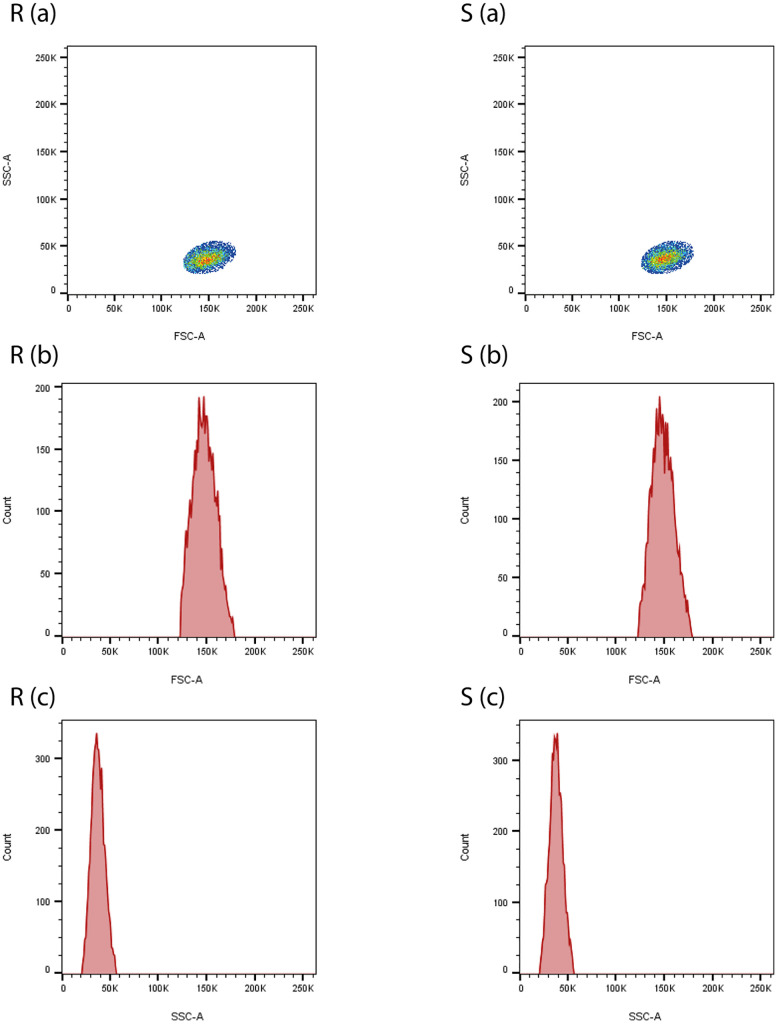
Comparison of real (R (a-c)) and synthetic (S (a-c)) gated monocyte clusters using common analytical-based flow cytometry visualisation techniques, including Pseudocolour dot plots and dimensional histograms. The synthetic cluster replicates the real cluster, appearing visually indistinguishable and demonstrating high accuracy in their reproduction.

The consistent replication of the monocyte cluster across both graphical visualisations underscores the robustness of our dual-analysis approach. Notably, while Fig 9 leverages dotplots and kernel-smoothed ridgeline histograms to highlight the visual and distributional agreement between the real and synthetic clusters, Fig 10 below reinforces these findings by presenting the same data in a native flow cytometry FCS analysis software format. This additional representation not only validates the reproducibility of our synthetic clusters within standard analytical frameworks but allows the synthetic cluster to be directly compared with conventional flow cytometry outputs. Overall, the visual parity between Figs 9 and 10 demonstrate that our approach yields synthetic clusters that are highly similar in both visual and distributional characteristics, providing a robust analytical platform suitable for broader adoption and integration in routine cytometric workflows.

The replication method effectively utilises the Rosetta-Routine as a second-pass tool to individually process and capture the statistical properties of the original cluster in differing orientations to generate individual Variance-determined dimensional cluster components. These dimension-based component clusters capture the specific distribution properties of one dimension determined by the alignment of the cluster before analysis. The reorientation of these dimensional component clusters to match the original clusters rotation along with subsequent combining creates an accurate replication of the original cluster. This novel method allows the synthetic generation of multiple simple clusters which come together to accurately create a realistic synthetic cluster with a distribution complexity much greater than the sum of its parts. Utilising the method above with real gated biological flow cytometry cell clusters shows that the method successfully reconstructs real monocyte clusters which closely match visually, statistically and distributionally.

## Conclusions

The Rosetta-Routine is a powerful and adjustable tool for determining the cluster input variables required to generate synthetic replicate clusters in the replication methodology. The Rosetta-Routine is built to robustly analyse and model complex cluster distributions but has several limitations (described within the method section) which highlight the need to utilise the system proficiently and with expertise to gain accurate results. Within this scope, we have demonstrated quantitative agreement between original and cluster replicates through variance and accuracy analyses, with visual and histogram-based comparisons that show preservation of cluster geometry and distributional characteristics.

Potential future improvements for the Rosetta-Routine could include implementing a more robust technique for determining the principal axis orientation to prevent possible ‘leakages’ of statistics between dimensions. Furthermore, opening up the model to be able to utilise other distributions, primarily exponential or log-normal cluster distributions, may expand the overall modelling capacity and method simplicity. Additionally, research into optimal methods for the ‘building up’ of datasets cluster by cluster into complete complex sample-like datasets with outliers and other typical sample characteristics is an avenue of subsequent work. Future work should also explore cross-laboratory and multi-operator validation to assess method generalisability.

This work presents a novel demonstration of the ability to analyse the statistical and distribution characteristics of a real live cell monocyte cluster data and build synthetic replicates that closely reproduce the structural and statistical characteristics of real data in practical flow cytometry workflows with a very high level of accuracy. The synthetic datasets generated should be interpreted as controlled, statistically equivalent realisations of the observed data rather than exact replicas. The extension to this work will be to extend the analysis to higher dimensionalities accompanied by orthogonal validation and cluster generation of a full FC dataset which includes lymphocytes, monocytes, granulocytes, beads, debris and noise.

## Materials and methods

### Ethics statement

The real flow cytometry data utilised in this study were secondary anonymised datasets used under ‘favourable’ ethical approval granted by the Loughborough University Ethics Committee. All ethical considerations and data handling procedures were conducted in accordance with the General Data Protection Regulation (2016) and the Human Tissue Act (2004).

**Fig 11:**
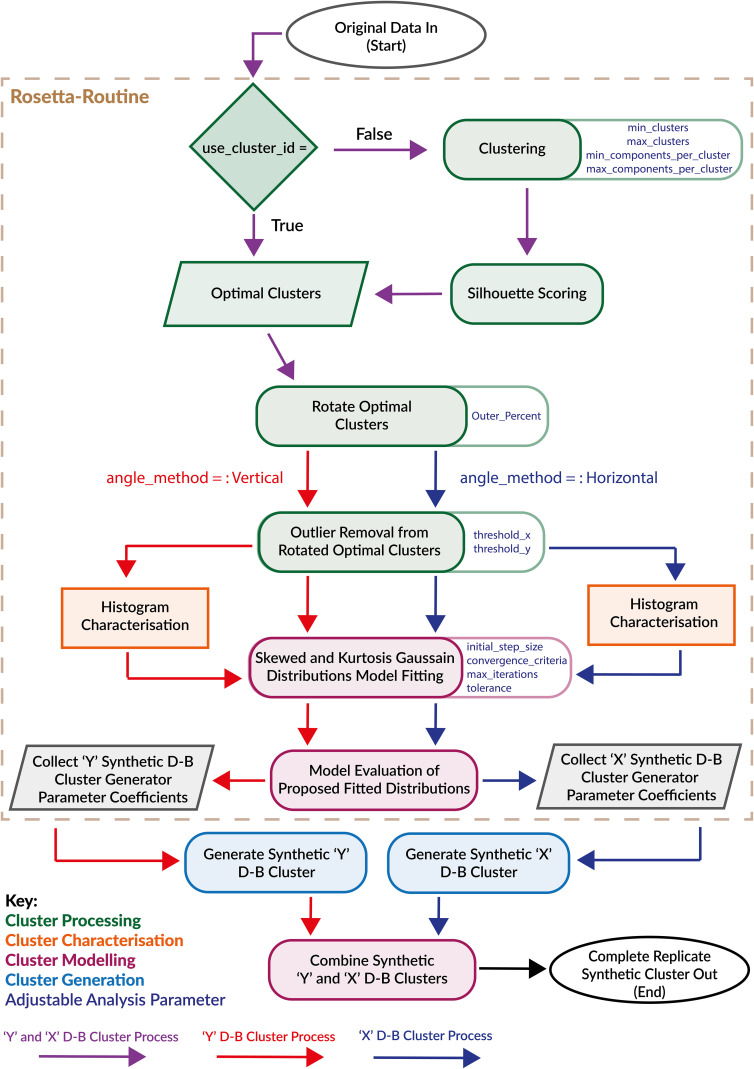
The replication methodology carried out to generate synthetic representations of real flow cytometry clusters made up of two key workflows. The first workflow is the Rosetta-Routine, which determines the CluGen cluster generator parameters for the proposed Dimensional-Based (D-B) synthetic clusters and carries out multiple cluster processing, characterisation and modelling functions to deduce these parameter values. The second workflow consists of how we use these values to generate synthetic cluster data to match the original real flow cytometry cluster.

The following replication methodology combines a blend of uniquely tailored cluster evaluation, processing, characterisation, and modelling techniques, containing multi-stage iterative refinement systems Fig 11. Enabling the determination of the precise input variables required to computationally construct statistically equivalent replicates of original cluster data using the CluGen synthetic cluster generator program [39].

The subsequent replication methodology sections explain the purpose, theoretical foundation, and rationale for each primary component of the synthetic cluster model parameter determination process for flow cytometry cell cluster data.

### Real flow cytometry data

The reference patient data used for generation and comparison studies with the synthetic data was provided with prior ethical approval by our collaborator, The United Kingdom National External Quality Assessment Service for Leucocyte Immunophenotyping (UK NEQAS-LI), with patient details robustly anonymised prior to being obtained. The datasets originated from stabilised testing samples previously collected for the ongoing ISO 17043 accredited UK NEQAS-LI CD34^+^ Stem Cell Enumeration Programme and contained unknown amounts of CD34^+^ cells [40–42]. All synthetic flow cytometry data was generated utilising the probabilistic terms derived from the robustly anonymised patient data and is agnostic in nature, and as such cannot be traced back to any individual.

### Determination of cluster generator input variables

#### Cluster evaluation protocol set up.

The Rosetta-Routine has multiple adjustable set up parameters to control differing operational aspects during the analysis of real cell clusters, Table 6. These features range from setting boundaries for clustering, easing or restricting the model fitting, and controlling the rotational methods applied to the optimal clusters. Furthermore, the ‘Use_cluster_ID*’* function enables the usage of unlabeled cluster data, manually gated clusters and pre-clustered CSV-formatted data.

**Table 6:** Initial adjustable analysis usage options and associated definitions for the Rosetta-Routine. Multiple adjustable usage options covering a wide range of processes throughout the Rosetta-Routine provide adaptable analysis approaches in cluster data processing.

Purpose	Parameter	Attribute type	Description
**Cluster Properties**	min_clusters	Integer	Minimum number of clusters to search for.
	max_clusters	Integer	Maximum number of clusters to search for.
	min_components_per_cluster	Tuple(Integer, Integer)	Lower limit of components in each dimension for each cluster (x, y).
	max_components_per_cluster	Tuple(Integer, Integer)	Upper limit of components in each dimension for each cluster (x, y).
	use_cluster_id	Boolean (true or false)	Boolean flag to determine whether to use cluster labels.
**Model Fitting**	max_iterations	Integer	Maximum number of model-fitting attempts.
	tolerance	Float	Model-fitting success tolerance.
**Rotation Adjustment**	angle_method	Symbol	Method for aligning cluster orientation (:Vertical or:Horizontal).
	outer_percent	Float	Percentage of outer points to omit temporarily in cluster rotation calculations.
**Outlier Removal**	threshold_x	Float	Threshold for outlier removal in the X dimension, in Standard Deviation units.
	threshold_y	Float	Threshold for outlier removal in the Y dimension, in Standard Deviation units.

### Optimal clustering and evaluation

The imported cluster data was subsequently evaluated through optimal clustering silhouette scoring metric implementations within the load_and_find_ clusters function of the Rosetta-Routine. Silhouette scoring is commonly utilised in cluster quality analysis [43]. Silhouette scores range from -1 to +1 and provide a powerful quantitative basis for selecting the ideal clustering configuration, thereby increasing the relative confidence in the cluster quality of the initial data.

**Fig 12:**
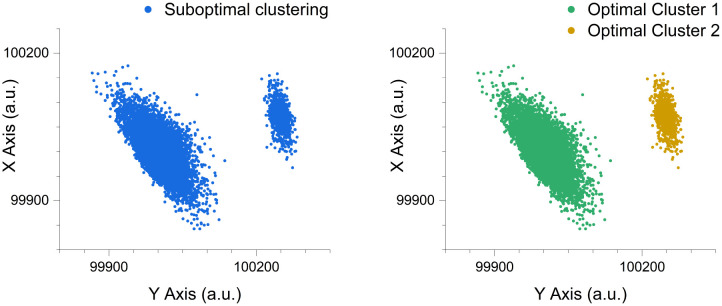
The Rosetta-Routine implementation of K-means clustering separates the original unlabeled suboptimal clustering into an optimised clustering arrangement. K-means clustering separates the original unlabeled suboptimal clustering (left), into an optimised clustering arrangement (right). The observed scenario scored a silhouette value of 0.79, indicating well-separated and distinct clusters. Data points are synthetically generated with axis labels representing arbitrary units (a.u.).

Determining the optimal number of clusters for a dataset can be achieved through various methods and algorithms, each offering differing insights into the dataset’s inherent structure. By default, the Rosetta-Routine utilises the standard K-means algorithm, a commonly applied unsupervised clustering method [44–47], Fig 12.

Additionally, silhouette scoring helped assess the statistical ‘purity’ of clusters, indicating if further refinement was necessary during later characterisation or model-fitting stages. If silhouette scores were low (<0.25), it indicated that the clustering results may be suboptimal, prompting the need to use alternative clustering techniques.

However, there is an option to bypass this built-in clustering and the subsequent evaluation scoring via the Rosetta-Routine’s use_cluster_id parameter. Setting this parameter’s Boolean flag attribute to true enables users to directly input pre-clustered data from a clustering method of their choice, allowing flexibility in integrating with a range of clustering methodologies and pre-labeled data.

### Standardised cluster orientations using principal axis alignment

Before cluster characterisation and modelling commenced, the optimal clusters previously identified underwent multiple pre-processing techniques. The aim of these pre-processing procedures was to ensure that the clusters are well-defined, aligned for consistent analysis and free from extreme outlier data points that could distort or reduce the efficiency and robustness of the modelling process.

Principal axis alignment processes reoriented and/or rotated entire clusters of data points clockwise to a common primary direction of maximum variability, or principal axis. Principal axis alignment of the optimal clusters simplified the subsequent Skew analysis, reducing orientation-based discrepancies and bias in the data analysis process.

**Fig 13:**
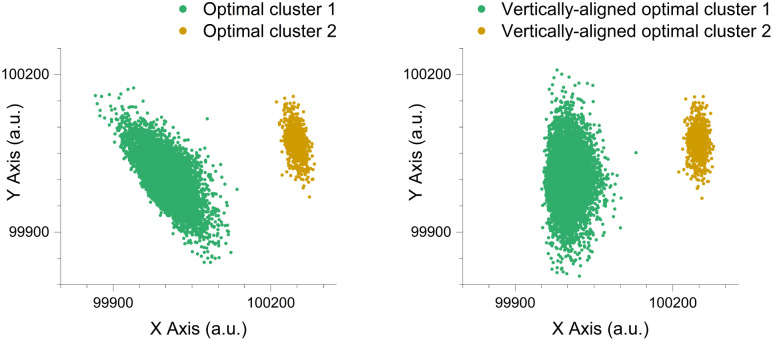
Comparison graphs showing the rotational vertical alignment of the optimal clusters based on their principal axis. Rotational vertical alignment of the optimal clusters (left), based on their principal axis (right). Data points are synthetically generated with axis labels representing arbitrary units (a.u.).

The Rosetta-Routine includes an angle_method function for rotation adjustment, allowing clusters to be aligned to the vertical orientation based on either their principal (longest:) or minor (shortest:) axis Fig 13.

The Outer_percent parameter enabled the temporary exclusion of an adjustable percentage of outermost points from the cluster before calculating the rotation for principal axis alignment. This process kept the impact of outlier-induced Skewing of the angle calculations to a minimum but ensured the primary core structural shape of the cluster was preserved. The full spatial transformation applied to each empirical cluster, specifically its centroid and principal-axis rotation, is recorded and the inverse of that transformation used to place the synthetic replicate clusters back into the original sample’s geometry when generated. This inverse-transform adjustment is applied deterministically for every cluster and restores the original relative geometry between clusters and thus preserves the biologically meaningful inter-cluster relationships within samples.

### Removal of skewed Gaussian model outliers in rotated optimal clusters

In Biology, data populations are often observed to follow a Gaussian-like distribution (high density central core which tapers into lower density edges). This is because biological variability, due to the fundamental differences among individual measurements, frequently create ‘bell-shaped’ distributions when measured across large populations. Treating cell-based cytometric clusters as Gaussian provides a robust first-order model that captures the dominant behaviour of many cell populations. Recognising, however, that real cytometry clusters (for example, monocyte, lymphocyte and granulocyte populations in CD34^+^ enumeration samples) are not perfectly normal and can display skewed tails and multimodal substructure. Our method incorporates higher-order distribution metrics, which aim to explicitly mitigate the principal risks of excessive feature loss associated with a strict normality assumption. Each empirical cluster is characterised not only by mean and covariance but also by skewness and kurtosis estimated along two approximately orthogonal axes. Furthermore, clusters may be represented as a weighted mixture of Gaussian components with distinct means, variances, skew parameters and relative weights through the utilisation of the min_components_per_cluster and max_components_per_cluster parameters. This hybrid strategy preserves the analytical convenience and numerical stability of the Gaussian components when modelling Gaussian-like clusters; while incorporating asymmetric tails and peripheral dispersion through skew estimation and component mixing of the initial first-order Gaussian models, yielding multivariate synthetic clusters that better retain both central compactness and realistic edge behaviour. We note that further gains in fidelity could be realised by incorporating additional distributional families (for example, heavy-tailed or copula-based models) and regard these extensions as avenues for future refinement.

Outlier removal is predicated on the common mathematical assumption that extreme values (± 3 Standard Deviations) within large approximately Gaussian-like distribution data are atypical, do not represent the primary statistical distribution and disproportionately influence parameter estimates. When the main goal is to characterise the predominant cell population accurately, including excessively extreme values can greatly compromise the real-life functionality of the model.

Application of a conservative cutoff to define the core of each cluster protects the first and higher moments (mean, variance, skewness, kurtosis) from being skewed by a small number of extreme events that lie relatively far from the dense cluster centre; without this trimming, such extremes can inflate and destabilise moment estimates and thereby propagate bias through the synthetic-generation pipeline. This initial trimming is not intended to discard genuine peripheral structure but is a minimal conservative trade-off to stabilise the core fit. Moreover, the modelling order and post-modelling adjustments are explicitly designed to better preserve overall fidelity while subsequently recovering realistic tail behaviour.

Precise utilisation of these controllable high-resolution methods to remove outlier values in-line with downstream objectives will ensure the integrity and core properties of a cluster’s distribution are preserved. While this process may slightly reduce the fidelity of the final, synthetic representation of the cell population relative to the raw data, it is crucial for achieving an appropriate and biologically relevant model of the underlying distribution.

In skewed Gaussian distribution modelling, outliers distort parameter estimates, especially the Mean, Standard Deviation, Skewness and Kurtosis which are essential for accurate model fitting. To address this, the Rosetta-Routine includes a robust permanent remove_outliers tool within the ‘Main’ function that utilises standard deviation units (z-scores) to identify outliers in each dimension.

**Fig 14:**
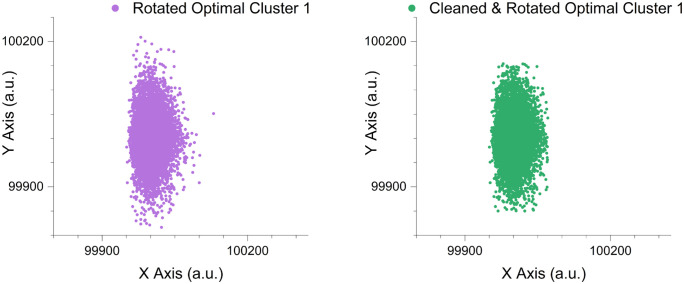
Comparison graphs showing the removal of outliers from the optimal cluster. The left graph shows the removal of points located over 5 Standard Deviations from the geometric mean, while the right graph shows the removal of points located over 3.5 Standard Deviations from the geometric mean in both individual dimensions. Data points are synthetically generated with axis labels representing arbitrary units (a.u.).

Threshold parameters (threshold_x and threshold_y) allow for adjustable sensitivity by excluding data points with z-scores exceeding the specified values, Fig 14. This dimension-specific thresholding of rotated optimal clusters increases tuning flexibility without excessive loss of data structure.

### Determination of the initial distribution parameters for Rosetta-Routine model fitting

Local histogram-characterised individual cluster statistics were used as the initial values for a conditional Expectation-Maximisation-based algorithmic model, which fit the desired number of components to each dimension of each optimal cluster through repetitive iterations until logical constraint-approved convergence was achieved. Bayesian information criterion scoring selected the ‘best’ optimal mixture model candidate provided by the Expectation-Maximisation algorithm, though a measure of trade-off between ‘goodness-of-fit’ and ‘simplicity’ for each dimensional distribution of each individual cluster.

**Fig 15:**
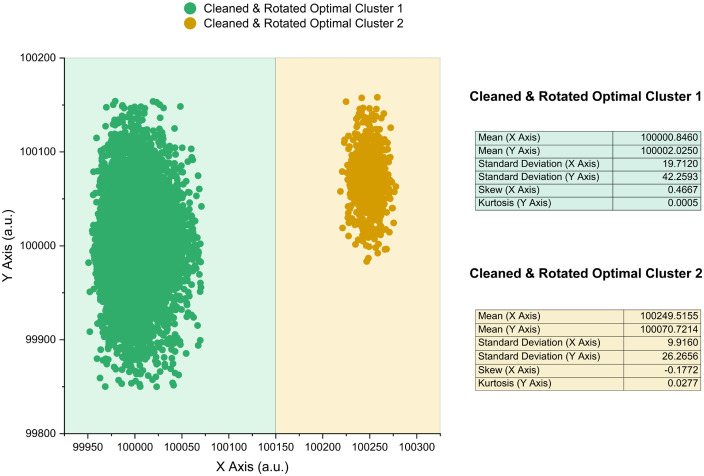
Histogram-based determination of the initial distribution parameters for the optimal cleaned rotated clusters before model-fitting. The green and yellow box overlays visually represent the number and locale for each calculated histogram bin, with the printed mathematical output for each cluster’s initial distribution parameter values per component displayed in the tables to the right of the graph. Data points are synthetically generated with axis labels representing arbitrary units (a.u.).

The initialise_parameters_from_histogram function was called before running the model fitting Expectation-Maximisation-based algorithm. The function calculated an initial set of parameter values (Mean, Standard Deviation, Skewness, Kurtosis) for each distribution component in each dimension of a cluster. The cleaned and rotated optimal cluster data underwent histogram analysis, with the data in each dimension divided into a number of bins equal to the respective number of specified components (Gaussain distribution peaks) in the corresponding dimension. Local peak identification within each histogram bin led to a mathematical-derived output of initial distribution values describing each peaks associated bin, Fig 15. These initial distribution parameter values aimed to represent the most significant features within the dataset, providing robust starting points for the Expectation-Maximisation-based algorithm to refine further.

These relatively simplistic histogram-based initialisation methods were chosen instead of implementing all model-fitting though maximum likelihood estimation primarily due to factors relating to computational cost and time efficiency. The later distribution fitting method requires the estimation of three separate parameters simultaneously for each component along with the possible need to fit multiple combinations of components for each cluster dimension. These features incur additional high computational costs within an already computationally intensive process if solely carrying out model fitting with maximum likelihood estimation. Instead, Histogram characterisation provides a beneficial middle ground approach to remove a portion of the computational cost (by stream-lining initial model fitting) but retain the accuracy of the final modelling results.

#### Cluster model fitting and evaluation.

To address the natural asymmetries frequently observed in real-world biological datasets, the synthetic clusters are modelled with incorporated measures of skewness and kurtosis within conventional Gaussian distributions. Each component within each dimension of a cluster is represented by a skewed-Gaussian distribution function with independently adjustable modelling parameters for the Mean (*μ*), Standard Deviation (*σ*), and Skewness (*α*) or Kurtosis values(*β*). In this work, we introduce two variations: the Skewed Gaussian X-Axis distribution and the Kurtosis Gaussian Y-Axis distribution. The Skewed Gaussian X-Axis distribution is defined by the following set of equations:


$$\begin{array}{c} fx(x;\mu ,\sigma ,\alpha )=\;\frac{\text{2}}{\sigma }\varphi (\text{𝓏})\Phi (\alpha \text{𝓏}) \end{array}$$ (1)

$$\begin{array}{c} \text{𝓏}=\frac{\text{𝓍}-\mu }{\sigma } \end{array}$$ (2)

$$\begin{array}{c} \varphi \left(\text{𝓏}\right)=\frac{\text{1}}{\sqrt{\text{2}\pi }}e^{\frac{-{\text{𝓏}}^{\text{2}}}{\text{2}}} \end{array}$$ (3)

$$\begin{array}{c} \Phi \left(\alpha \text{𝓏}\right)=\frac{\text{1}}{\text{2}}(\text{1}+\text{erf}(\frac{\text{𝒶𝓏}}{\sqrt{\text{2}}})) \end{array}$$ (4)

Here, Equation (1) represents the probability density function (PDF) for the skewed distribution along the X-axis, with Equation (2) defining the standardised variable (*z)*. Equation (3) is the standard normal PDF, and Equation (4) modifies the cumulative distribution function (CDF) to incorporate Skewness through the parameter *α*. Custom distribution type structures for the skewed Gaussian X-Axis provide enhanced flexibility for generating asymmetrical distributions, with *μ* as the location parameter, *σ* as the scale parameter, and *α* as the shape parameter. The Skewed Gaussian Y-Axis distribution is defined by the following set of equations:


$$\begin{array}{c} fY\left(\text{𝓎};\mu ,\sigma ,\beta \right)=\;\frac{\text{2}}{\sigma }\varphi (\text{𝓏})\Phi (\beta \left|\text{𝓏}\right|) \end{array}$$ (5)

$$\begin{array}{c} \text{𝓏}=\frac{\text{𝓎}-\mu }{\sigma } \end{array}$$ (6)

$$\begin{array}{c} \varphi \left(\text{𝓏}\right)=\frac{\text{1}}{\sqrt{\text{2}\pi }}e^{\frac{-{\text{𝓏}}^{\text{2}}}{\text{2}}} \end{array}$$ (7)

$$\begin{array}{c} \Phi (\beta \left|\text{𝓏}\right|)=\frac{\text{1}}{\text{2}}(\text{1}+\text{erf}(\frac{\beta \left|\text{𝓏}\right|}{\sqrt{\text{2}}})) \end{array}$$ (8)

Equation (5) provides the PDF for the Kurtosis Normal Y-Axis distribution. Equations (6) and (7) denote the standardisation and the standard normal PDF, respectively. In Equation (8), the absolute value (∣*z*∣) is employed in the CDF to ensure that the measure of tail extremity is symmetric, capturing the Kurtosis effect through the shape parameter (*β)*. This design allows the distribution to model the extremity of both tails without directional bias.

The kurtosis function differs from the Skew function with its focus on the tail behaviour of the distribution rather than its asymmetry. While Skewness, with measured influence in the X-axis distribution, captures the asymmetry of the data and is directional, Kurtosis, modelled in the Y-axis distribution, measures the “tailedness” or extremity of the distribution’s tails. Because Kurtosis concerns the overall tail behaviour without regard to direction, the data skewing effect is symmetric, capturing the extremity of both tails without bias towards one direction. In contrast, the X-axis Skew distribution does not require such an absolute deviation because Skewness is sensitive to whether the data is relatively skewed to the ‘left’ or ‘right’. The X axis skew and Y axis Kurtosis of each cluster is jointly modelled within the Expectation-Maximisation algorithm (detailed below) due to translational requirements when mapping to CluGen functions. This integrated approach ensures possible Skewness-induced Kurtosis in perpendicular axes is accounted for when model fitting. Thereby, enhancing the accuracy and relevance of the final synthetic clusters.

### Expectation-Maximisation-based algorithm for parameter optimisation

**Fig 16:**
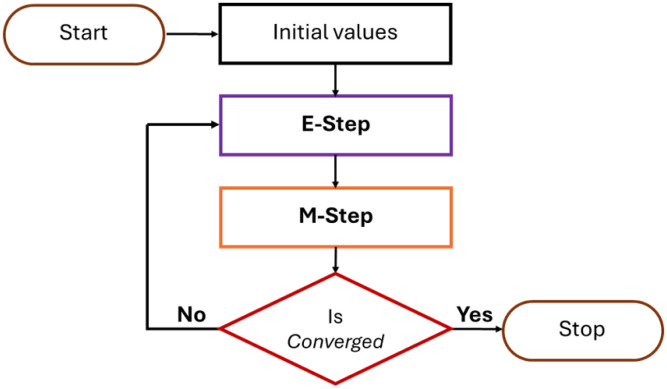
Expectation-Maximisation-based algorithm for parameter optimisation. The Expectation-Maximisation algorithm first calculates the probability that each data point belongs to each component of the singular or mixture-based model (E-step). It then ensures the parameters of each component of the model are updated to maximise the likelihood function (M-step). Once parameter changes between iterations are below a specified tolerance threshold then convergence is satisfied, and the algorithm terminates.

Local histogram-characterised statistics of each individual cluster were used as the initial parameter estimates for a constrained Expectation-Maximisation-based algorithmic joint skewed Gaussian mixture modelling function. This algorithm fit the desired number of components to each dimension of every optimal cluster through repetitive iterations until logical constraint-approved convergence was achieved (see **Fig 16**) or the limit for the maximum number of fitting attempts was reached. Each iteration consisted of two main steps: the expectation step and maximisation step.

During the expectation step, the algorithm computes the responsibilities for the current model, specific to the current values of the model’s adjustable parameters (*μ*, *σ*, *α* or *β*). The responsibilities of the model are gradings of the probabilities that each individual data point belongs to each component (and / or cluster) within the model. The mathematical assumptions of the expectation step are derived from Bayesian probability theory [48].

The *μ*, *σ* and *α* or *β* parameters are subject to additional adjustable logical constraints and numerical stability techniques uncommon with traditional expectation maximisation algorithms, **Table 7**. These additional processes enhance both the robustness and accuracy of the parameter measurements to ensure usage relevance to the CluGen cluster generator.

**Table 7:** Additional adjustable logical constraints and numerical stability techniques applied to the *μ*, *σ*, *α* and *β* distribution parameters during the expectation-maximisation algorithm.

Feature	Objective	Justification
Joint dimensional responsibility calculation	Joint responsibility calculation of X and Y for each component.	Ensures that dependencies between dimensions are accounted for in the final probability assignment.
Mean update step size regulation	Mean update is constrained with a 5% step size limit per iteration.	Restricts large parameter jumps, improving stability and preventing oscillatory behaviour.
Handling of X-dimension skewness	Final X-dimension skew values are divided by π and 2	Corrects for 2D-to-1D mapping in cluster generation, ensuring accurate scaling with CluGen’s bimodal method of generating clusters.

The final check of model convergence is achieved when the extent at which all the individual parameters (*μ*, *σ*, *α* and *β*) change between iterations is less than the defined tolerance attribute. This then halts the algorithm as further iterations are deemed unlikely to yield significant improvements. Achieving an appropriate convergence of parameters ensures that the resultant synthetic cluster models are stable and can reliably replicate the real data’s statistical and distributional properties.

### Evaluating model-fitting quality of the proposed synthetic cluster distributions

The Rosetta-Routine methodology uses the Bayesian Information Criterion (BIC) [49] as the evaluation metric to assess the quality of the refined cluster distribution model. This process enables internal benchmarking of the proposed model to ensure an accurate representation of the original data’s underlying structure.

The model, which is produced as the output of the Expectation-Maximisation algorithm, is evaluated to determine whether the current configuration is optimal. A major advantage of BIC scoring is its innate ability to balance model fit with complexity, thereby helping to prevent overfitting. Models with an excessive number of components are penalized under BIC, favoring configurations that achieve the best trade-off between “goodness-of-fit” and “simplicity.”

The BIC scoring step after Expectation-Maximisation refinement is crucial because it delivers a statistically grounded approach for selecting an optimal probability distribution model. Additionally, BIC scoring is relatively simple, enabling the entire process to be very computationally and temporally efficient. Making BIC scoring suitable for iterative model selection assessments across differing component configurations and scenarios. Once scoring is complete, the configuration with the overall lowest full model BIC is chosen as the optimal cluster modelling solution. This configuration serves as the foundation for the subsequent synthetic data generation methods, with the relevant cluster generator input variables retrieved from the Rosetta-Routine analysis output files (S4 File).

### Increasing the complexity of synthetically generated clusters

Greater distributionally complex synthetic clusters were generated using a novel Principal axis and skewness-kurtosis based covariance (PASKC) method. The PASKC method utilised the Rosetta-Routine as a second-pass tool to perform operations (similar to the initial stages of PCA) to individually process and capture the statistical properties of the original cluster in the vertical and horizontal orientations to generate individual Principal-axis-determined dimensional cluster output coefficients for CluGen. These resultant vertical and horizontal orientated cluster coefficients were utilised to generate corresponding dimensional-based clusters (D-B). Both vertical and horizontal D-B clusters were then rotated and overlaid to form one complete synthetic cluster.

### Real vs. Synthetic cluster validation

Statistical equivalence and distributional comparisons between the real and synthetically generated clusters were carried out via histogram and statistical analysis. In addition, modelling and analytical cross validation of the real cluster compared to the synthetic replicant was completed and visualisation of the real and synthetic clusters within specialised flow cytometry software to demonstrate user-usage scenarios was accomplished.

### *In silico* data modeling and analysis software

All *In silico* studies were carried out on the same workstation running a 64-bit windows operating system. All code writing, data modelling and computational simulations were performed using the Julia programming language (v 1.9.1), with code development and debugging carried out in Visual Studio Code (v 1.93.1). All subsequent analysis was performed using OriginPro, Version 2024 (10.1.0.178). The synthetic clusters were generated using initial software obtained from the GitHub repository ‘CluGen.jl’ (V1.0.0) by Nuno Fachada & Diogo de Andrade (2023) (https://github.com/clugen/CluGen.jl). Additional novel code along with a list of the required package dependencies required in the Julia programming language are provided in S2 and S3 Files.

#### Practical considerations and limitations of the Replication Method.

The method for determining the cluster generator input variables (Rosetta-Routine) for synthetic data generation is a robust framework, which models complex skewed-and-kurtosis-Gaussian distributions. However, the method does have several practical considerations and limitations in generating accurate replicates of synthetic clusters. These features are related to multiple aspects of the process such as workload requirements, sensitivity, data quality, and cluster shape assumptions.

The Rosetta-Routine involves computationally intensive processing steps such as Silhouette Scoring and the Expectation-Maximisation-driven model refinement. These processes are often taxing on computing resources, with computational demand scaling with the size of the dataset and number of clusters and/or components. Specifying excessively restricting parameters controlling the iterative refinement techniques can increase the runtime significantly. Higher specification operating systems may be required when dealing with excessively large or multiple datasets in this manner. However, appropriate fine-tuning of parameters such as the convergence_criteria will ensure an ideal trade-off between the accuracy of results and overall program runtime.

The Rosetta-Routine methodology relies on several user-specified parameters such as, Outer_Percent or initial_step_size. The values defined within these parameters directly influence results. Default ‘blanket’ parameter settings may not optimally analyse all types of datasets equally. Requiring users to manually adjust and balance multiple parameters, which can be challenging without prior experience or domain knowledge.

The Rosetta-Routine currently assumes all individual clusters can be modelled using skewed-gaussian distributions. This limitation in available probability distribution functions means that the synthetic clusters can successfully replicate a wide range of elliptical and moderately asymmetric phenotypes but may not capture highly irregular multimodal shapes or continuous transitional gradients between populations effectively. Increasing the Rosetta-Routine’s modelling capabilities through the inclusion of additional distribution families is a subject of further work. Additionally, it is recognised that the generation of complete biological flow cytometry samples would most likely require the implementation of heavy-tailed or non-parametric samplers to replicate common sample features exhibiting extreme heavy-tailing, often seen in artefactual populations such as debris events. Furthermore, the primary focus of the Rosetta-Routine is to replicate clusters which are elliptical or spherical in nature and as a result need to exhibit a clear definable direction. This is due to the method’s reliance on directionality analysis through principal axis alignment. As a result, the Rosetta-Routine’s current reliance on principal-axis alignment further biases it toward clusters with a definable directionality (elliptical or spherical shapes), and can underperform when moment extraction is unstable because clusters are diffuse or lack clear definition.

Excessive magnitudes in cluster skew typically (>0.2) or kurtosis results (>0.5) returned from the Rosetta-Routine in an X or Y cluster component may require additional processing to achieve valid results. High values of Skew or Kurtosis overwhelm the Rosetta-Routine Skewed-Gaussian distribution modelling probabilities, resulting in the generation of clusters with a non-realistic harsh flat edge.

However, cluster morphologies with these truncated ‘flat edges’ which adhere to the directionality requirements may also be valid targets for synthetic cluster generation but usage in this research has been limited. Furthermore, the generation of clusters with a ‘curved’ principal axis is beyond the scope of this methodology, requiring additional extensive ‘workarounds’ to overcome the built-in linear support line restrictions of the CluGen software. The generation of highly curved clusters may be better implemented with another cluster generator with included Bezier curve cluster generating functionalities [25].

Many of these limitations are not unique to this work but are intrinsic to cluster-based parametric modelling more generally. To mitigate practical risk, the Rosetta-Routine implements transparent fit-quality metrics and per-cluster diagnostics that flag when assumptions are being stretched, supporting informed use and preventing silent model failure. Where diagnostic indicators suggest poor parametric fit, users are advised to treat generated outputs with caution or to employ alternative modelling strategies. Looking forward, planned extensions include expanding the pool of candidate distribution families, and integrating non-parametric samplers and multicomponent strategies that can reproduce multimodality, heavy tails and non-clustered background gradients. Finally, while current implementations focus on two-dimensional cluster representations for algorithmic clarity and reproducibility, scaling to fully multivariate, higher-dimensional generative models is a priority for future development to enable more detailed biological comparisons and more faithful whole sample synthesis.

## Supporting information

S1 File Cluster Data (SI-1 Cluster Data.xlsx). Contains all the datasets used for figure generation and quantitative analyses in the manuscript, including the real and synthetic event-level measurements used for population modelling.
(XLSX)

S2 File Cluster Generating Julia Script Example (SI-2 cluster generator script.jl). Provides an example Julia script used to generate the synthetic cell cluster data.
(ZIP)

S3 File Rosetta-Routine Script Example (SI-3 Rosetta-Routine script.jl). Shows an example of the full Rosetta-Routine pipeline script, used for generational variance modelling and synthetic cluster replication.
(ZIP)

S4 File Rosetta-Routine Analysis Results (SI-4 Rosetta-Routine generational variance analysis results.xlsx). Contains the generational variance dataset outputs used to validate the synthetic cluster replication fidelity of the Rosetta-Routine analysis.
(XLSX)
